# Reserve and Maintenance in the Aging Brain: A Longitudinal Study of Healthy Older Adults

**DOI:** 10.1523/ENEURO.0455-21.2022

**Published:** 2022-02-02

**Authors:** Epifanio Bagarinao, Hirohisa Watanabe, Satoshi Maesawa, Kazuya Kawabata, Kazuhiro Hara, Reiko Ohdake, Aya Ogura, Daisuke Mori, Noritaka Yoneyama, Kazunori Imai, Takamasa Yokoi, Toshiyasu Kato, Shuji Koyama, Masahisa Katsuno, Toshihiko Wakabayashi, Masafumi Kuzuya, Minoru Hoshiyama, Haruo Isoda, Shinji Naganawa, Norio Ozaki, Gen Sobue

**Affiliations:** 1 Department of Integrated Health Sciences, Nagoya University Graduate School of Medicine, Nagoya, Aichi 461-8673, Japan; 2 Brain & Mind Research Center, Nagoya University, Nagoya, Aichi 466-8550, Japan; 3 Department of Neurology, Fujita Health University School of Medicine, Toyoake, Aichi 470-1192, Japan; 4 Department of Neurology, Nagoya University Graduate School of Medicine, Nagoya, Aichi 466-8550, Japan; 5 Department of Neurosurgery, Nagoya University Graduate School of Medicine, Nagoya, Aichi 466-8550, Japan; 6 Department of Community Healthcare and Geriatrics, Nagoya University Graduate School of Medicine, Nagoya, Aichi 466-8550, Japan; 7 Department of Radiology, Nagoya University Graduate School of Medicine, Nagoya, Aichi 466-8550, Japan; 8 Department of Psychiatry, Nagoya University Graduate School of Medicine, Nagoya, Aichi 466-8550, Japan; 9 Aichi Medical University, Nagakute, Aichi 480-1195, Japan

**Keywords:** aging, brain, healthy adults, maintenance, MRI, reserve

## Abstract

The aging brain undergoes structural changes even in very healthy individuals. Quantifying these changes could help disentangle pathologic changes from those associated with the normal human aging process. Using longitudinal magnetic resonance imaging (MRI) data from 227 carefully selected healthy human cohort with age ranging from 50 to 80 years old at baseline scan, we quantified age-related volumetric changes in the brain of healthy human older adults. Longitudinally, the rates of tissue loss in total gray matter (GM) and white matter (WM) were 2497.5 and 2579.8 mm^3^ per year, respectively. Across the whole brain, the rates of GM decline varied with regions in the frontal and parietal lobes having faster rates of decline, whereas some regions in the occipital and temporal lobes appeared relatively preserved. In contrast, cross-sectional changes were mainly observed in the temporal-occipital regions. Similar longitudinal atrophic changes were also observed in subcortical regions including thalamus, hippocampus, putamen, and caudate, whereas the pallidum showed an increasing volume with age. Overall, regions maturing late in development (frontal, parietal) are more vulnerable to longitudinal decline, whereas those that fully mature in the early stage (temporal, occipital) are mainly affected by cross-sectional changes in healthy older cohort. This may suggest that, for a successful healthy aging, the former needs to be maximally developed at an earlier age to compensate for the longitudinal decline later in life and the latter to remain relatively preserved even in old age, consistent with both concepts of reserve and brain maintenance.

## Significance Statement

Aging is associated with gray matter (GM) decline, yet some individuals tend to remain cognitively healthy even in advanced age. What differentiates the brains of “healthy agers” from those individuals who are prone to faster cognitive decline still remains unclear. Using longitudinal magnetic resonance imaging (MRI) data from a carefully selected cohort, we examined the brain aging characteristics of healthy agers. Our findings showed that, even in this population, frontal-parietal regions have faster longitudinal rate of GM decline, whereas some temporal-occipital regions appeared relatively preserved. These findings may suggest that, for a successful healthy aging, frontal-parietal regions need to be maximally developed to compensate for the longitudinal decline later in life and the temporal-occipital regions to remain relatively preserved even in old age.

## Introduction

The aging brain undergoes several structural changes even in healthy individuals. Quantifying these changes could help dissociate pathologic cases from those associated with the normal aging process. Several studies have investigated cross-sectional age-related changes in brain volumes (Good et al., 2001; Taki et al., 2004; Allen et al., 2005; Grieve et al., 2005; Smith et al., 2007; Bagarinao et al., 2018). However, the extent to which these cross-sectional changes represent actual changes in brain volumes in individuals remains controversial (Pfefferbaum and Sullivan, 2015). Cross-sectional aging studies, specifically those involving the lifespan, could be confounded by several factors including secular changes because of differences in living condition, nutrition, medical care, and other environmental factors, making it difficult to differentiate these changes from those directly attributed to aging.

Longitudinal aging studies (Resnick et al., 2000, 2003; Rusinek et al., 2003; Scahill et al., 2003; Raz et al., 2005; Fjell et al., 2009; Takao et al., 2012; Taki et al., 2013; Pfefferbaum et al., 2013; Storsve et al., 2014; Gracien et al., 2017; Vinke et al., 2018) avoid some of these problems. With each individual participant serving as their own control, longitudinal studies are more suitable in quantifying the extent of age-related brain volume changes in individuals and are effective in separating the effect of secular changes in brain volumes occurring over multiple generations in a population. Methods to analyze longitudinal images have also advanced (Ashburner and Ridgway, 2012; Reuter et al., 2012), leading to significant improvement in detecting subtle within-individual volumetric changes.

Evidence from both cross-sectional and longitudinal aging studies have clearly demonstrated that aging is associated with gray and white matter (WM) volume reductions in older adults. These reductions are not uniform throughout the whole brain (Resnick et al., 2003; Raz et al., 2005; Fjell et al., 2009). Specifically, frontal regions, which are crucial for higher-order cognitive functions, are shown to be more vulnerable to the aging process exhibiting faster rates of decline than others (Raz et al., 1997; Resnick et al., 2003; Fjell et al., 2009; Pfefferbaum et al., 2013). In addition, the rate of decline may also depend on age showing acceleration in older individuals (Raz et al., 2005; Fjell et al., 2009). Despite this, some individuals tend to remain cognitively healthy even in advanced age. What differentiates the brains of “healthy agers” from those individuals who are prone to faster cognitive decline still remains unclear. Concepts such as cognitive/brain reserve (Stern, 2002; Satz et al., 2011; Barulli and Stern, 2013) or brain maintenance (Nyberg et al., 2012) have been advanced to explain this discrepancy. Under these hypotheses, individuals with greater reserve or well maintain functional or structural brain integrity in old age are less vulnerable to cognitive decline. But direct anatomic evidence supporting these hypotheses remained very limited.

In this study, we examined age-related changes in the brain of healthy older adults to uncover potential mechanisms that differentiates the brains of healthy agers. We hypothesized that brains of healthy agers have aging characteristics that are consistent with the reserve and maintenance hypotheses. For this, we used longitudinal magnetic resonance imaging (MRI) data from a carefully selected cohort of elderly participants with age ranging from 50 to 80 years old at baseline scan who remained medically and cognitively healthy throughout the study period to identify the relevant changes associated with healthy aging. We leveraged on recent improvements in longitudinal data analysis to quantify longitudinal rate of change in total GM and WM volumes as well as identify regional pattern of age-related atrophic changes across the whole brain in these participants. We also used linear mixed effects (LME) models to simultaneously account for both the cross-sectional and longitudinal components of our data. To make later comparisons easier, we also employed established longitudinal analysis pipeline from FreeSurfer and used readily available brain parcellation for regional volumetric analysis.

## Materials and Methods

### Participants

MRI data from 227 participants (male/female = 74/153) were included in the analysis. Participants were carefully selected from a pool of volunteers participating in our on-going aging cohort study (Bagarinao et al., 2018) using the following inclusion criteria: (1) age at baseline scan from 50 years old and above; (2) cognitively normal with Mini-Mental State Examination (MMSE) score (Folstein et al., 1975) equal to 26 and above and Addenbrooke’s Cognitive Examination–Revised (ACE-R) total score (Mioshi et al., 2006; Dos Santos Kawata et al., 2012) equal to 89 and above for all visits during the study; (3) with no observable abnormality in the brain (e.g., asymptomatic cerebral infarction, benign brain tumor, leptomeningeal cyst, etc.) examined using MRI; and (4) no obvious brain atrophy or WM abnormalities characterized by hyperintensities in T2-weighted images that were grade 2 or 3 based on the Fazekas hyperintensity rating system. All participants had three longitudinally acquired MRI data with mean inter-scan intervals of 1.08 years (range: 0.75–2.66) from the baseline scan to the first follow-up and 1.06 years (range: 0.91–1.59) from the first follow-up to the second follow-up. All MR images were examined by two Japanese certified neurologists (H.W., K.H.) and a neurosurgeon (S.M.) for anatomic abnormalities. The participants’ characteristics at baseline are summarized in Table 1.

**Table 1:** Participants’ characteristics at baseline scan

Age group (M/F)		All mean (SD)	Male mean (SD)	Female mean (SD)
50s (15/47)	Age, years Age range, years	54.65 (3.07) 50.17–59.92	54.38 (3.43) 50.17–59.92	54.73 (2.98) 50.17–59.08
	MMSE	29.52 (0.67)	29.33 (0.82)	29.57 (0.62)
	ACE-R	97.42 (1.50)	97.13 (1.55)	97.51 (1.49)
	Education, years	14.03 (2.25)	15.00 (3.40)	13.72 (1.66)
	BDI	4.56 (5.33)	2.53 (2.50)	5.21 (5.83)
60s (24/59)	Age, years Age range, years	65.21 (2.73) 60.17–69.92	65.28 (2.59) 60.42–69.67	65.18 (2.81) 60.17–69.92
	MMSE	29.43 (0.77)	29.50 (0.72)	29.41 (0.79)
	ACE-R	96.53 (2.46)	97.00 (2.62)	96.34 (2.39)
	Education, years	13.55 (2.09)	14.75 (2.27)	13.07 (1.82)
	BDI	4.18 (4.46)^*^	2.65 (3.31)^*^	4.78 (4.72)
70s (35/47)	Age, years Age range, years	73.25 (2.64) 70.08–79.67	73.42 (2.71) 70.42–79.67	73.13 (2.60) 70.08–79.50
	MMSE	28.98 (1.02)	28.91 (1.09)	29.02 (0.97)
	ACE-R	94.70 (2.86)	94.34 (3.02)	94.96 (2.74)
	Education, years	13.17 (2.22)	14.31 (2.26)	12.32 (1.78)
	BDI	5.15 (4.62)	4.29 (4.09)	5.79 (4.92)

* One participant has no data.

M, male; F, female; MMSE, Mini-Mental State Examination total score; ACE-R, Addenbrooke’s Cognitive Examination–Revised total score; BDI - Beck Depression Inventory score.

The study conformed to the Ethical Guidelines for Medical and Health Research Involving Human Subjects endorsed by the Japanese Government and was approved by the Ethics Committee of Nagoya University Graduate School of Medicine. All participants provided written informed consent before joining the study.

### MRI

All selected participants underwent at least three MRI sessions at the Brain & Mind Research Center, Nagoya University using a Siemens Magnetom Verio (Siemens) 3.0 T scanner with a 32-channel head coil. For each imaging session, a high-resolution T1-weighted MR image was acquired for each participant using a three-dimensional magnetization prepared rapid acquisition gradient echo (MPRAGE, Siemens) pulse sequence (Mugler and Brookeman, 1990) with the following acquisition parameters: repetition time (TR)/MPRAGE TR = 7.4/2500 ms, echo time (TE) = 2.48 ms, inversion time (TI) = 900 ms, 192 sagittal slices with a distance factor of 50%, 1-mm thickness, and in-plane voxel resolution of 1.0 × 1.0 mm^2^, field of view (FOV) = 256 mm, flip angle (FA) = 8 degrees, and total scan time equal to 5 min and 49 s.

### Image preprocessing

The acquired T1-weighted MR images were automatically preprocessed using the longitudinal preprocessing pipeline (Reuter et al., 2012) in FreeSurfer (Dale et al., 1999; Fischl and Dale, 2000; Fischl et al., 2002), which is documented and can be freely downloaded online (http://surfer.nmr.mgh.harvard.edu/). The analysis workflow is divided into three stages (Reuter et al., 2012). In the first stage, all images were independently preprocessed using the default FreeSurfer analysis pipeline, which includes, among others, subcortical segmentation and cortical surface parcellation, based on FreeSurfer’s integrated atlases, of the input images. In the second stage, an unbiased template for each participant was created using images from all time points. The generated templates were also similarly processed using the default analysis pipeline. In the final stage, the images from all time points for each participant were registered and resampled into the unbiased participant-specific template space and then ran through FreeSurfer’s analysis pipeline but using the information generated in the second stage (template processing) to initialize the relevant processing algorithms. This is to ensure that the images from all time points for a given participant were processed under the same initial condition to reduce processing variability as well as improve robustness and sensitivity (Reuter et al., 2012). The generated data from the last stage were used in the succeeding analyses.

As part of its preprocessing pipeline, FreeSurfer performs subcortical segmentation and cortical parcellation of the input images using included atlases, where several measures including gray matter (GM) volume are automatically extracted. Here, we only examined age-related changes of volumetric data. Specifically, the total GM volume, total WM volume, total CSF volume, and estimate of the total intracranial volume (eTIV), as well as cortical and subcortical volumes were obtained from the longitudinally processed images (last stage). For cortical volumes, we used the cortical parcellation based on the Desikan–Killany atlas (Desikan et al., 2006), which is readily available and fully integrated in FreeSurfer. For subcortical regions, we obtained volumes of several regions of interest (ROIs) including amygdala, caudate, hippocampus, pallidum, putamen, and thalamus, as well as the cerebellar cortex and WM volumes.

### Experimental design and statistical analyses

To model age-related changes in volumes, we used LME models. Specifically, we examined the following three LME models:

(1) $${\text{Vol}}_{\text{ij }}={\text{ a}}_{0}+{\text{ a}}_{1}{\text{ bAge}}_{\text{i }}+{\text{ a}}_{2}{\text{ bTime}}_{\text{ij }}+{\text{ a}}_{3}{\text{ Sex}}_{\text{i }}+{\text{ a}}_{4}{\text{ eTIV}}_{\text{i }}+{\text{ b}}_{\text{i}0}+{\text{ ε}}_{\text{ij}}$$

(2) $${\text{Vol}}_{\text{ij }}={\text{ a}}_{0}+{\text{ a}}_{1}{\text{ bAge}}_{\text{i }}+{\text{ a}}_{2}{\text{ bTime}}_{\text{ij }}+{\text{ a}}_{3}{\text{ Sex}}_{\text{i }}+{\text{ a}}_{4}{\text{ eTIV}}_{\text{i }}+{\text{ b}}_{\text{i}0}+{\text{ b}}_{\text{i}1}{\text{ bTime}}_{\text{ij }}+{\text{ ε}}_{\text{ij}}$$

(3) $${\text{Vol}}_{\text{ij }}={\text{ a}}_{0}+{\text{ a}}_{1}{\text{ bAge}}_{\text{i }}+{\text{ a}}_{2}{\text{ bTime}}_{\text{ij }}+{\text{ a}}_{3}{\text{ Sex}}_{\text{i }}+{\text{ a}}_{4}{\text{ eTIV}}_{\text{i }}+{\text{ a}}_{5}{\text{ bAge}}_{\text{i}}\text{*}{\text{ bTime}}_{\text{ij }}+{\text{ b}}_{\text{i}0}+{\text{ b}}_{\text{i}1}{\text{ bTime}}_{\text{ij }}+{\text{ ε}}_{\text{ij}}$$

In the above equations, Vol_ij_ represents volume (total GM, WM, or CSF as well as regional volumes) for the *i*th participant at scan *j*, the a_i_s are the coefficients associated with the fixed effects terms (observed effects common to all participants), the b_i0_ and b_i1_ represent coefficients associated with the random effects terms (observed effects unique to the trajectory of each individual participant), bAge_i_ represents age at baseline scan modeling the cross-sectional component of the data, bTime_ij_ represents the time interval between baseline scan and follow-up scans (0 for images at baseline scan) modeling the longitudinal component of the data, Sex_i_ represents the participant’s sex encoded as 0 for men and 1 for women, eTIV_i_ represents the estimated intracranial volume, and ε_ij_ is the error term. Equation 1 allows individual variations in the intercept (b_i0_), whereas Equation 2 allows variations in both the intercept (b_i0_) and slope (b_i1_) associated with time from baseline (bTime_ij_). Equation 3 takes into account the interaction between baseline age and time from baseline (bAge_i_ * bTime_ij_) as well as variations in both intercept and slope. We only included a linear term for bTime since there were only three longitudinal time points. Although previous lifespan studies have shown nonlinear relationship between age and brain volumes (Fjell et al., 2013; Pfefferbaum et al., 2013), we only considered a linear term in bAge in this study since the range of age covered was limited.

To identify the best fit model for the data, we used the likelihood ratio test (LRT) for nested models. First, we compared models 1 and 2 and tested the null hypothesis that the data are generated by model 1, the simpler model. If the null hypothesis cannot be rejected (*p*-value of the LRT, *p*_LRT_ > 0.05), model 1 wins; otherwise (*p*_LRT_ < 0.05), model 2 wins. The winning model is then compared with model 3. The best fit model was selected from the result of the second comparison testing the null hypothesis that the data are generated by the winning model of the first comparison, i.e., first comparison’s winning model if the second comparison’s *p*_LRT_ > 0.05 or model three if *p*_LRT_ < 0.05. For total volumes (GM, WM, and CSF), the best fit model was used as the final model. For regional volumes, we used a common model for all regions investigated. For this, we first obtained the best fit model for each regional volume using the LRT as described, and then selected a final model that included terms from the best fit models for all regions. Finally, we also performed independent analyses for each sex. For this, model parameters were estimated using the same LME model identified using all participants but excluding the term associated with sex. We used the functions *fitlme* and *compare*, both available in MATLAB (R2020a, MathWorks), for all LME analyses and for the LRT, respectively. To account for multiple comparisons when analyzing regional volumes, we used false discovery rate (FDR) corrected *p*-values to evaluate the statistical significance of the estimated parameters of the final model.

## Results

### Changes in total brain volumes

**Figure 1.**
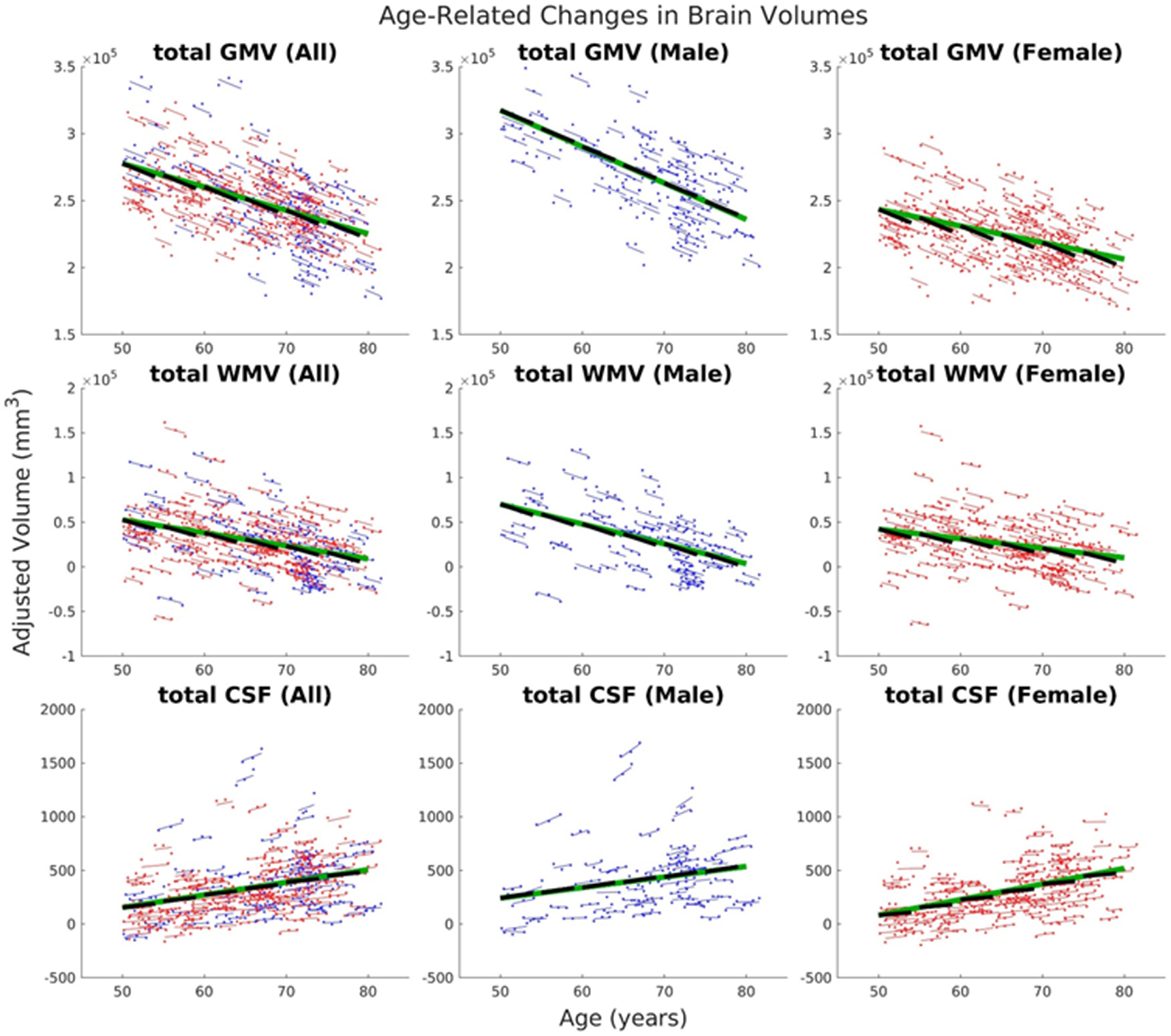
Age-related changes in total GM volume (first row), total WM volume (second row), and total CSF volume (third row). The first column showed LME model estimates using all participants’ data, the second column using only that of male participants, and the third column for female participants. Dots correspond to raw volumes adjusted for sex and eTIV. Colored thin lines are conditional fit of the longitudinal data using the estimated LME model. Longer green thick lines represent association with baseline age, whereas shorter black thick lines represent association with time for the longitudinal component. Data from male participants are shown in blue, whereas that of female participants are shown in red. Estimated model parameters, *t* values, and *p*-values are given in Extended Data Figure 1-1.

For total GM and WM volumes, model 1 was identified as the best fit model (for model 1 vs 2 comparisons, *p*_LRT_ = 0.64 and 0.37, respectively, and for model 1 vs 3, *p*_LRT_ = 0.47 and 0.12, respectively). For total CSF volume, model 2 was identified as the best fit model (for model 1 vs 2 comparison, *p*_LRT_ = 0.02 and for model 2 vs 3, *p*_LRT_ = 0.19). Using model 1 as the final LME model for both total GM and WM, model parameters were estimated. We observed significant (*p* < 0.05) decreases in total GM volume as a function of age for both within (longitudinal component) and across (cross-sectional component) individuals (Fig. 1, top row). The longitudinal rate of decline (a_2_ = −2497.54 mm^3^ per year) was relatively higher (more negative) than the cross-sectional component (a_1_ = −1748.90 mm^3^ per year) when all participants were included in the analysis. Separately, men showed faster cross-sectional rate of decline compared with women, but the longitudinal rate was almost the same in both sexes. The longitudinal component was also higher compared with the cross-sectional component in women, but was the same in men. In terms of the total WM volume, we observed the same behavior as that of the total GM volume (Fig. 1, middle row). Total WM volume also decreased with age in both cross-sectional (a_1_ = −1456.70 mm^3^ per year) and longitudinal (a_2_ = −2579.83 mm^3^ per year) components, with a faster rate of decline in the cross-sectional component in men as compared with women. For the total CSF volume, we used model 2 as the final LME model. The total CSF volume increased with age (Fig. 1, bottom row). Both the longitudinal and cross-sectional components have almost the same value in the analysis where men and women were separated or combined. Estimated LME parameter values, including *t* and *p*-values, are given in Extended Data Figure 1-1.

10.1523/ENEURO.0455-21.2022.f1-1 Extended Data Figure 1-1 Estimated model parameters and their corresponding *t* values and *p*-values of the optimal LME models for the total GM volume, total WM volume, and total CSF volume using data from all participants, male only participants, and female only participants. Download Figure 1-1, XLS file.

### Changes in cortical volumes

**Figure 2.**
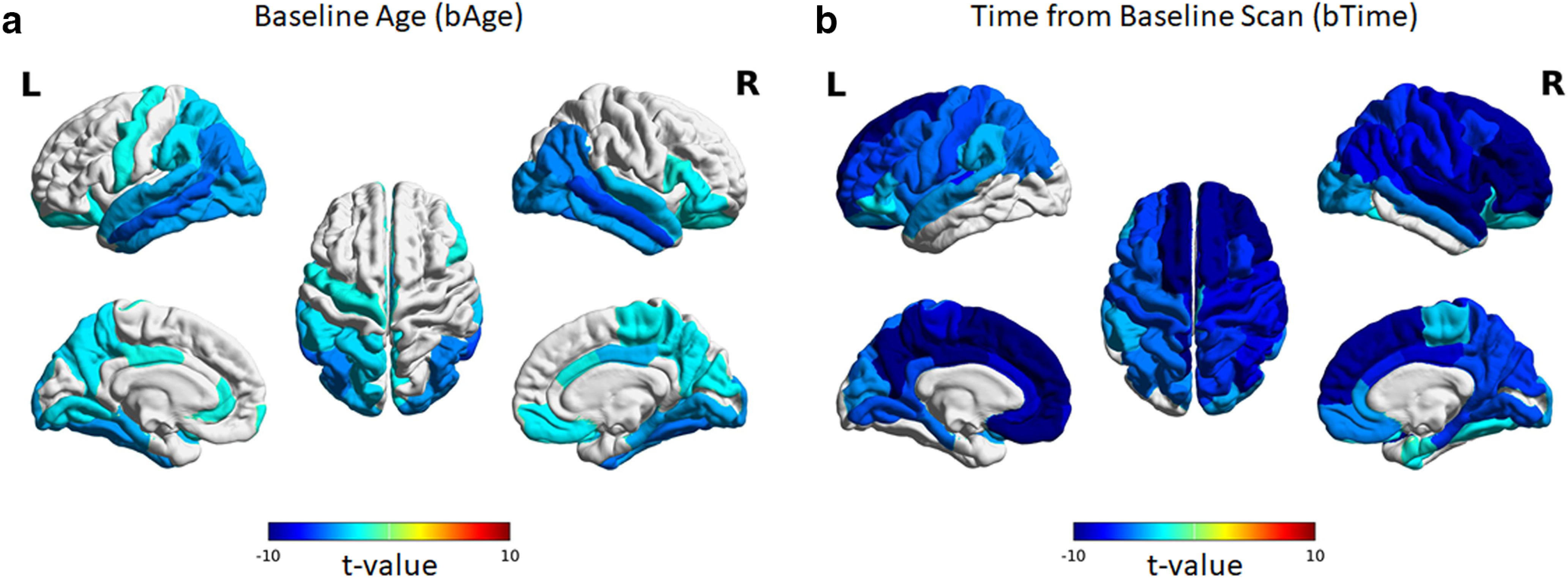
Linear relationship between (***a***) regional GM volumes and baseline age as well as (***b***) regional GM volumes and time from baseline scan. The cortex is divided into 34 ROIs for each hemisphere based on the Desikan–Killany atlas. Estimated model parameters, *t* values, and *p*-values are given in Extended Data Figures 2-1, 2-2, 2-3 using data from all participants, male only, and female only, respectively.

Age-related volumetric changes in regional GM were examined using the Desikan–Killany atlas consisting of 34 ROIs per hemisphere. The results are shown in Figure 2. Of the 68 ROIs, the best fit model for 51 ROIs (75%) was model 1 whereas that of the remaining 17 (25%) was model 2. For consistency throughout the whole brain, we used model 2 as the final LME model for all ROIs and recomputed the model parameters. Of the 68 ROIs, 36 showed significant (FDR q < 0.05) negative linear relationship with baseline age (Fig. 2*a*). These ROIs were mostly located in the occipital, parietal, and temporal lobes. The right inferior parietal ROI showed the highest rate of cross-sectional decline (a_1_ = −55.891 mm^3^ per year), followed by the right middle temporal ROI (a_1_ = −54.757 mm^3^ per year), then the left middle temporal ROI (a_1_ = −51.720 mm^3^ per year), right lateral occipital ROI (a_1_ = −49.624 mm^3^ per year), and left inferior parietal ROI (a_1_ = −46.979 mm^3^ per year), among others. The rate of cross-sectional decline was also not symmetric across hemispheres and varied from ROI to ROI.

10.1523/ENEURO.0455-21.2022.f2-1 Extended Data Figure 2-1 Estimated model parameters and their corresponding *t* values and *p*-values (corrected for multiple comparisons using FDR) of the optimal LME models for regional GM volume using all participants’ data. Download Figure 2-1, XLS file.

10.1523/ENEURO.0455-21.2022.f2-2 Extended Data Figure 2-2 Estimated model parameters and their corresponding *t* values and *p*-values (corrected for multiple comparisons using FDR) of the optimal LME models for regional GM volume using data from male participants only. Download Figure 2-2, XLS file.

**Figure 3.**
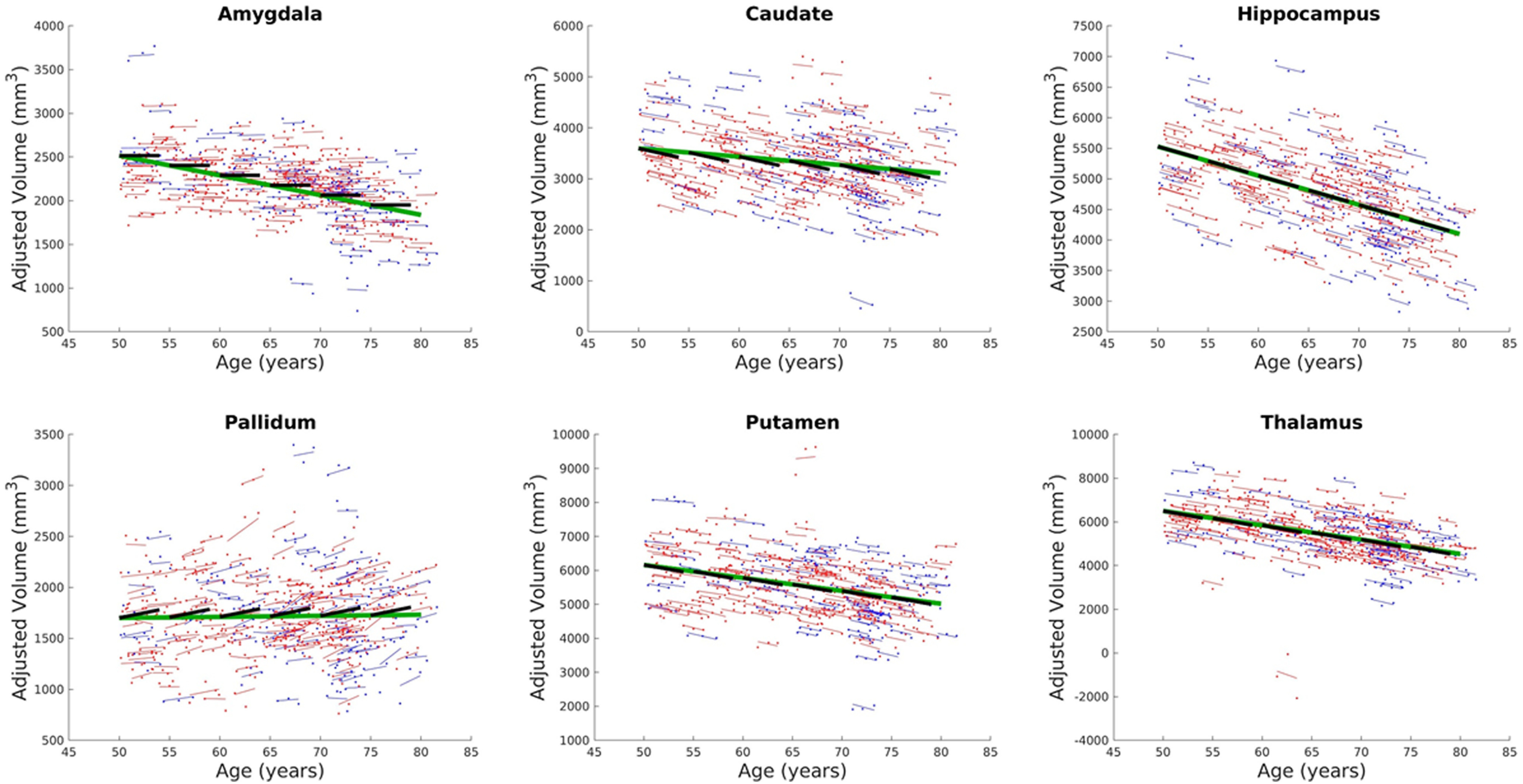
Estimated LME models for selected subcortical ROIs using all participants’ data. Volumes are adjusted for sex and eTIV. Dots correspond to adjusted raw volumes. Colored thin lines are conditional fit of the longitudinal data using the estimated LME model. Longer green thick lines represent association with baseline age, whereas shorter black thick lines represent association with time for the longitudinal component. Data from male participants are shown in blue, whereas data from female participants are shown in red. Estimated model parameters, *t* values, and *p*-values are given in Extended Data Figure 3-1.

10.1523/ENEURO.0455-21.2022.f2-3 Extended Data Figure 2-3 Estimated model parameters and their corresponding *t* values and *p*-values (corrected for multiple comparisons using FDR) of the optimal LME models for regional GM volume using data from female participants only. Download Figure 2-3, XLS file.

In terms of longitudinal change, more widespread decreases in cortical volumes were observed (Fig. 2*b*). Specifically, 58 of the 68 ROIs showed significant (FDR q < 0.05) negative linear relationship with time relative to baseline scan. The left superior frontal ROI showed the highest rate of longitudinal decline (a_2_ = −208.082 mm^3^ per year), followed by the right superior frontal (a_2_ = −168.871 mm^3^ per year), then the right rostral middle frontal (a_2_ = −110.260 mm^3^ per year), the right precentral (a_2_ = −106.436 mm^3^ per year), and the right inferior parietal (a_2_ = −86.818 mm^3^ per year), among other ROIs. Similar to the relationship with baseline age, the rate of decline was also not uniform across ROIs and not symmetric across hemispheres with the right hemisphere showing relatively more ROIs with significant decline in GM volume and higher rates than the left. The ten ROIs that did not show significant (FDR q > 0.05) longitudinal decline included the left bank of the superior temporal sulcus, left entorhinal, left fusiform, left inferior temporal, left lateral occipital, left middle temporal, bilateral temporal pole, right isthmus cingulate, and right pericalcarine. The parameter estimates, *t* values, and FDR-corrected *p*-values for all ROIs are given in the Extended Data Figures 2-1, 2-2, 2-3 for the analysis including all participants, men only, and women only, respectively.

### Changes in volumes of selected subcortical regions

We also examined age-related decline in selected subcortical regions using LME model 2 (Fig. 3). We used LME model 2 to be consistent with the ROI-based analyses. GM volume in amygdala showed significant (FDR q < 0.05) negative linear relationship with baseline age (a_1_ = −22.604 mm^3^ per year), but its relationship with time relative to baseline scan was not significant. For caudate, both the cross-sectional component (a_1_ = −16.194 mm^3^ per year) and longitudinal component (a_2_ = −43.376 mm^3^ per year) were significant (FDR q < 0.05) with the latter exhibiting a higher rate of decline compared with the former. The hippocampus showed significant negative linear relation with both baseline age (a_1_ = −47.696 mm^3^ per year) and time (a_2_ = −46.656 mm^3^ per year) and with almost the same rate of decline. Similarly, the putamen and thalamus also showed significant negative relationship (FDR q < 0.05) with baseline (a_1_ = −38.047 mm^3^ per year and a_1_ = −65.520 mm^3^ per year, respectively) and time (a_2_ = −50.924 mm^3^ per year and a_2_ = −82.332 mm^3^ per year, respectively) with the latter slightly higher than the former. On the other hand, pallidum showed significant (FDR q < 0.05) increase with time from baseline (a_2_ = 19.647 mm^3^ per year), but its change with baseline age was not significant (FDR q > 0.05). Estimated LME parameters including *t* values and FDR-corrected *p*-values are given in Extended Data Figure 3-1.

### Changes in cerebellar volumes

**Figure 4.**
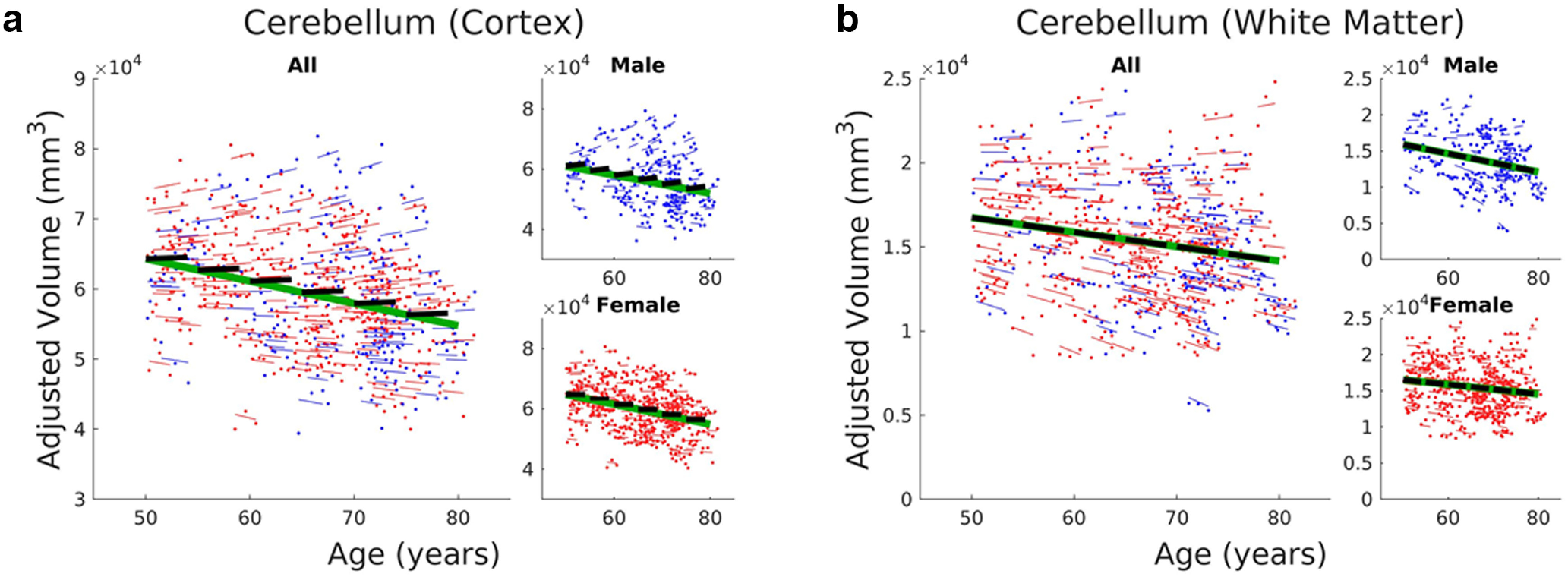
Estimates of LME model for cerebellar volumes: (***a***) cortex and (***b***) WM. Sex-specific LME estimates are also shown in the insets. Dots correspond to raw volumes adjusted for sex and eTIV. Colored thin lines are conditional fit of the longitudinal data using the estimated LME model. Longer green thick lines represent association with baseline age, whereas shorter black thick lines represent association with time for the longitudinal component. Data from male participants are shown in blue, whereas that of female participants are shown in red. Estimated model parameters, *t* values, and *p*-values are also given in Extended Data Figure 3-1.

Age-related cerebellar volume changes are summarized in Figure 4 using LME model 2. For the cerebellar cortex, we observed significant (FDR q < 0.05) cross-sectional decrease with age (a_1_ = −318.091 mm^3^ per year); however, the longitudinal change was not significant (Fig. 4*a*). This is also true for the analyses involving only men or women (Fig. 4*a*, insets). The latter showed a relatively higher rate of cross-sectional decline (a_1_ = −331.446 mm^3^ per year) compared with the former (a_1_ = −301.831 mm^3^ per year). In contrast, the cerebellar WM volume showed significant (FDR q < 0.05) negative linear relation with both baseline age (a_1_ = −86.161 mm^3^ per year) and time from baseline (a_2_ = −87.498 mm^3^ per year). The rates of decline were also the same. The rate of decline were also the same in both cross-sectional and longitudinal components when men and women were separately analyzed, although only the cross-sectional component in men was significant. They also exhibited faster rates of decline in both longitudinal and cross-sectional components than women. Estimated LME parameters are given in Extended Data Figure 3-1.

10.1523/ENEURO.0455-21.2022.f3-1 Extended Data Figure 3-1 Estimated model parameters and their corresponding *t* values and *p*-values (corrected for multiple comparisons using FDR) of the optimal LME models for selected subcortical ROIs using data from all participants, male only participants, and female only participants. This extended data supports both Figures 3, 4. Download Figure 3-1, XLS file.

## Discussion

Using a very healthy cohort of participants, we examined age-related changes in global as well as regional brain volumes using LME models that simultaneously account for both the cross-sectional (across participants) and longitudinal (within individuals) components of the data. Our results showed that first, age-related cross-sectional and longitudinal declines were observed in total GM and WM volumes. In contrast, total CSF volume increased with age. Second, age-related GM volume loss across the whole brain was not uniform, but also nonrandom. Longitudinal decline was more widespread and more prominent in the frontal and parietal regions and with the right hemisphere showing generally faster decline than the left. In contrast, cross-sectional changes were somewhat limited with the affected ROIs located mainly in the temporal and occipital regions. Third, longitudinal rates of decline were not fully captured by cross-sectional rates in this very healthy cohort, leading to an interesting pattern of GM decline across the whole brain. Specifically, in many frontal and parietal regions, significant GM decline was observed within individuals, but not across participants. In contrast, a number of temporal and occipital ROIs have GM volumes that declined across participants but not within individuals. Overall, these findings suggest that even in very healthy older adults, frontal-parietal regions declined with age in individuals, whereas some temporal-occipital regions appeared to be relatively preserved. By quantifying these changes, our estimates could provide baseline information when dissociating pathologic brain changes from changes driven by the normal aging process.

### Age-related changes in global volumes

Considering only the longitudinal effect as modelled by bTime, we obtained the rate of tissue loss of the total GM volume equal to 2.5 cm^3^ per year in older adults. This is about the same as that obtained previously in a longitudinal study involving participants with age ranging from 59 to 85 at baseline (Resnick et al., 2003), which highly overlapped in age with our cohort. Our estimate of the rate of WM loss (2.6 cm^3^ per year) was also close to the previous estimate of 3.1 cm^3^ per year (Resnick et al., 2003) and 2.4 cm^3^ per year for the very healthy elderly (Resnick et al., 2003). Since our participants represented a relatively very healthy cohort, our estimate is very close to the latter.

We also observed differences in cross-sectional rate, modelled by bAge, of total GM and WM loss between men and women with the former exhibiting faster rate of decline than the latter. Similar findings have been previously reported (Good et al., 2001). However, our result showed that the longitudinal rates of GM decline were about the same in both sexes after accounting for differences in TIV. This may suggest that the observed differences in the estimated cross-sectional rates may be mostly driven by cohort differences, rather than by actual differences in individual rate of decline. We do note that given the unbalanced number of men and women in our cohort, this finding may need further validation.

### Longitudinal decline and relative preservation characterized age-related changes in regional brain volumes

Differences in longitudinal (a_2_) and cross-sectional (a_1_) estimates of regional rates of GM decline were apparent across the whole brain. Longitudinally, the rate of GM volume decline was not uniform. Many ROIs in the frontal lobe (e.g., superior frontal, rostral middle frontal, right precentral, etc.) had most of the highest rate of GM decline, whereas those in the occipital lobe (e.g., left fusiform, lateral occipital, etc.) were relatively preserved, consistent with the findings of a previous longitudinal study (Vinke et al., 2018). Similar greater decline in frontal and parietal, as compared with temporal and occipital, has also been reported (Resnick et al., 2003; Pfefferbaum et al., 2013). To explain this selective vulnerability, several hypotheses have been proposed including the “last-in, first-out” principle, where GM increases during development is mirrored with GM decline during aging, observed mainly in transmodal regions (Douaud et al., 2014).

Despite the observed individual GM declined in these regions, no significant difference in GM volume were observed across participants. The implication is that the older participants in this very healthy cohort may have an initially higher GM volume in these regions at an earlier age to compensate for the observed individual rate of decline. This observation is consistent with the concept of reserve, which posits the accumulation of neural resources early in life (and presumably even at old age) to mitigate the effects of decline caused by aging (Cabeza et al., 2018). Frontal and parietal regions are associated with higher-order cognitive processes (Fedorenko et al., 2013; Yoon et al., 2017), are involved in the brain’s integrative functions as transmodal regions responsible for integrating information from unimodal regions (Mesulam, 1998), and are also late to mature during development, making these regions more amenable to the development of reserve capabilities. Pfefferbaum and colleagues (Pfefferbaum et al., 2013) have shown that changes in GM volumes in these regions can be better approximated using a cubic trajectory, where slower changes occur in-between inflection points. Douaud and colleagues (Douaud et al., 2014) also demonstrated that transmodal regions exhibit an inverse U-shape association with age, which could be amenable to factors influencing reserve via mechanisms that could heightened the curve’s peak value or shift its peak location at a later age. In fact, differences in peak values and peak onsets have been demonstrated between sexes with a higher and slightly later peak observed in women than in men (Douaud et al., 2014). Given this, maximum development (higher peak value) or delayed peak onset of frontal and parietal regions may be critical in achieving a cognitively healthy aging.

While many ROIs showed significant longitudinal GM volume loss, some temporal and occipital ROIs as well as amygdala and cerebellar cortex exhibited preservation of GM volumes relative to time from baseline scan. Temporal and occipital regions are known to mature early in development and late to atrophy. Thus, it is reasonable to assume that differences in volume in these regions among healthy agers are better explained by cohort-related effects as indicated by our result. In contrast to frontal-parietal regions, this finding therefore may suggest that the maintenance of normal general cognitive functioning could also be influenced by the relative preservation of these regions in older adults. In this respect, the concept of brain maintenance may aptly apply. Unlike reserve, this concept refers to the preservation of neural resources, via repair processes, to counteract decline regardless of the initial level of neural measures (Nyberg et al., 2012; Cabeza et al., 2018).

### Age-related changes in subcortical regions

In terms of subcortical regions, we observed significant (FDR q < 0.05) longitudinal GM decline in all regions examined except amygdala, pallidum, and the cortex of the cerebellum, consistent with previous reports (Fjell et al., 2013). Interestingly, the hippocampus, the thalamus, cerebellar WM, and somewhat the putamen have cross-sectional rates of change that were mostly consistent with their respective longitudinal rates of change. This is intriguing considering the observed differences in cross-sectional and longitudinal estimates in cortical regions in this cohort. Volumes in these subcortical regions can therefore be well-approximated from cross-sectional results. For amygdala, the observed relative volume preservation within individuals, but not across participants where the volume declined, was consistent with previous findings (Pfefferbaum et al., 2013). Similar cross-sectional GM decline were also reported in several other studies (Allen et al., 2005; Curiati et al., 2009; Fjell et al., 2013). On the other hand, the pallidum longitudinally increased with age, the only region exhibiting such behavior and is somewhat difficult to explain. However, the observed increase was mostly driven by women (Extended Data Fig. 3-1). In a large cross-sectional study (Potvin et al., 2016), bilateral pallidum has also been shown to exhibit a cubic trajectory across the lifespan, where relative increases in GM volume where observed within the age range of our cohort and particularly in women.

### Limitations

This study has limitations. Differences in sex were not fully explored because of the unbalanced number of men and women in our cohort. To account for this, sex was included as a covariate in most analysis and separate analyses for each sex were also performed. The age range is also limited from 50 to 80 years old. Thus, the linear model used in this study is limited within this age range. Finally, although cross-sectional and longitudinal rates of change in GM volume did not agree in most of the regions examined because of the way participants were selected (bias toward very healthy individuals), we were able to delineate the two effects by using LME models that simultaneously account for both. In the process, we were able to identify regions with GM volume that still significantly decline with age as well as regions with relatively well-preserved GM volumes even in the very healthy cohort.

## Conclusion

In conclusion, using LME models to dissociate cross-sectional and longitudinal changes associated with healthy aging, we quantify the rates of volumetric changes in the brain of older adults. Regional patterns of GM loss showed that regions maturing later in development are more vulnerable to longitudinal changes, whereas those that fully mature in the early stage are affected only by cross-sectional changes in healthy older cohort. This suggests that for a successful healthy aging, the former needs to be maximally developed at an earlier age to compensate for the longitudinal changes later in life and the latter to remain relatively preserved even in old age, consistent with both concepts of reserve and brain maintenance. Moreover, our findings could also provide the essential information for the rate and regional pattern of age-related decreases in total and regional GM volumes against which pathologic changes can be evaluated.

## Synthesis

Reviewing Editor: Christopher Colwell, UCLA School of Medicine Decisions are customarily a result of the Reviewing Editor and the peer reviewers coming together and discussing their recommendations until a consensus is reached. When revisions are invited, a fact-based synthesis statement explaining their decision and outlining what is needed to prepare a revision will be listed below. The following reviewer(s) agreed to reveal their identity: NONE. Note: If this manuscript was transferred from JNeurosci and a decision was made to accept the manuscript without peer review, a brief statement to this effect will instead be what is listed below.

The ms has been significantly revised and prior concerns about overlap in text addressed (iThenticate used to quantify this overlap_.

The resulting ms will make a nice contribution to the literature.
